# Supplementary material for: Targeting ITGβ3 to Overcome Trastuzumab Resistance through Epithelial–Mesenchymal Transition Regulation in HER2-Positive Breast Cancer
Source: Int J Mol Sci. 2024 Aug 8;25(16):8640. doi: 10.3390/ijms25168640 (PMC11354641; doi:10.3390/ijms25168640)
Supplement: Supplementary file 1 [file ijms-25-08640-s001.zip › ijms-3156158-supplementary.pdf]

Supplemantary

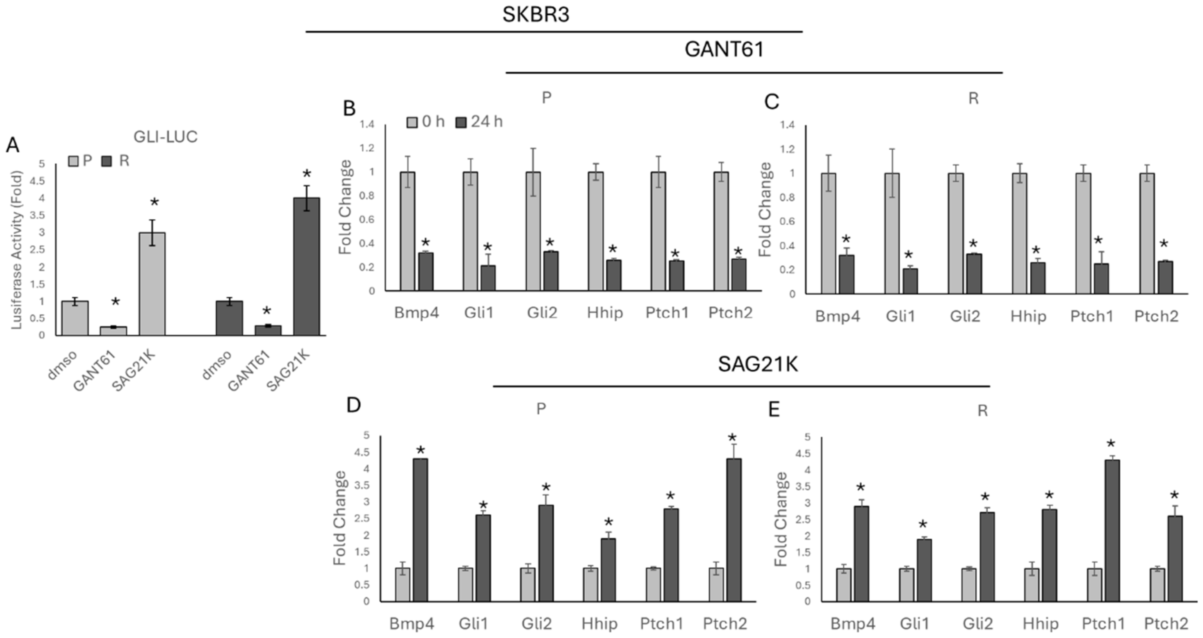

Supplementary Figure S1: GANT61 decrease hedgehog signalling while SAG21K increase in parental and resistant SKBR3, A) GAN61 is an hedhehog inhibitor and decreased Hedgehog activity, SAG21K is an hedhehog activator and increased Hedgehog activity in Lusiferase Reporter assay, B) GANT61 decrease hedgehog signalling responsive gene expressions in 24 h in parental cells, C) GANT61 decrease hedgehog signalling responsive gene expressions in 24 h in resistant cells, D) SAG21K increase hedgehog signalling responsive gene expressions in 24 h in parental cells, C) SAG21K increase hedgehog signalling responsive gene expressions in 24 h in resistant cells in SKBR3 cells. Student t test were used. * $p \leq 0.05$, n = 3 ± SD.

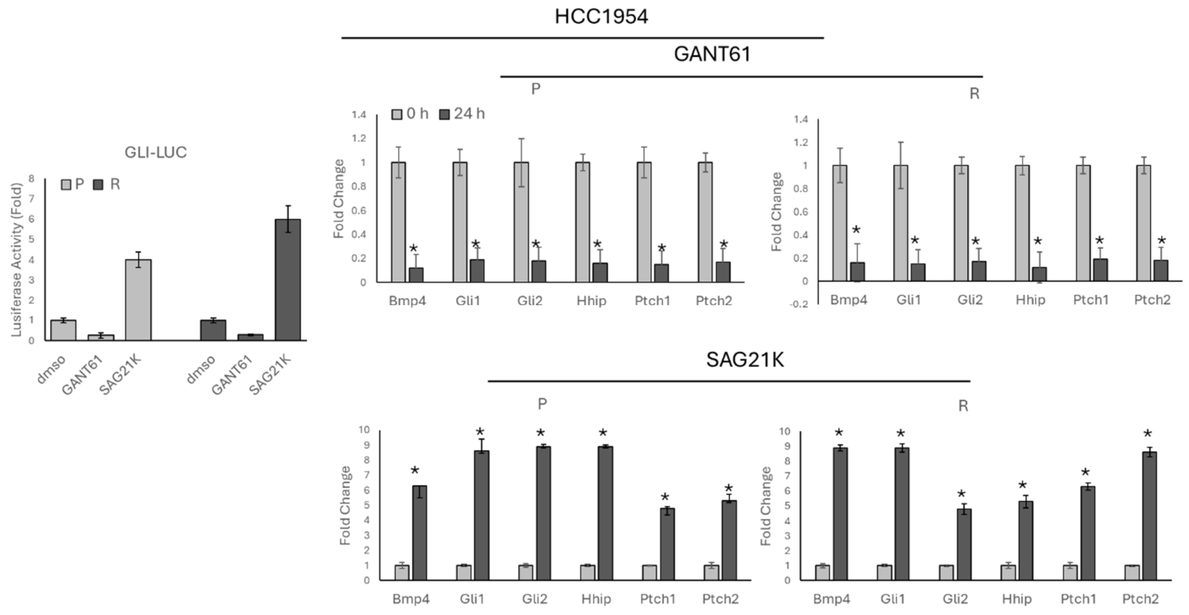

Supplementary Figure S2; GANT61 decrease hedgehog signalling while SAG21K increase in parental and resistant HCC1599, A) GAN61 is an hedhehog inhibitor and decreased Hedgehog activity, SAG21K is an hedhehog activator and increased Hedgehog activity in Lusiferase Reporter assay, B) GANT61 decrease hedgehog signalling responsive gene expressions in 24 h in parental cells, C) GANT61 decrease hedgehog signalling responsive gene expressions in 24 h in resistant cells, D) SAG21K increase hedgehog signalling responsive gene expressions in 24 h in parental cells, C) SAG21K increase hedgehog signalling responsive gene expressions in 24 h in resistant cells in HCC1599 cells. Student t test were used. * p ≤ 0.05, n = 3 ± SD.

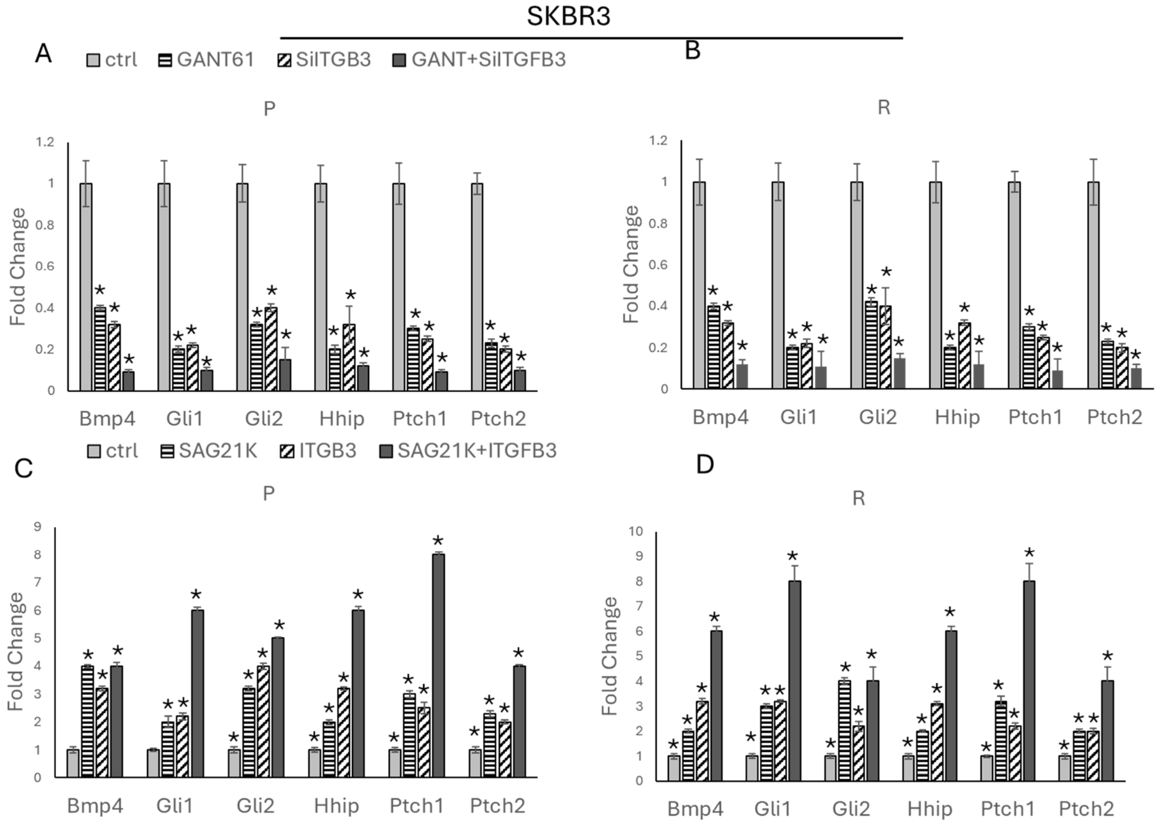

Supplementary Figure S3: Silencing of ITGB3 in the presence of GANT61 create a cumulative decrease while overexpression of ITGB3 in the presence of SAG21K create a cumulative increase in hedgehog responsive gene expressions in SKBR3 cells. A) Hedhehog responsive gene expressions analysed in the presence of GANT61 and/or ITGB3 silence in SKBR3 parental cells, B) Hedhehog responsive gene expressions analysed in the presence of GANT61 and/or ITGB3 silence in SKBR3 resistant cells C) Hedhehog responsive gene expressions analysed in the presence of SAG21K and/or ITGB3 overexpression in SKBR3 parental cells, D) Hedhehog responsive gene expressions analysed in the presence of SAG21K and/or ITGB3 overexpression in SKBR3 resistant cells. A two-way ANOVA variation test and Tukey post hoc test were used. n=3, * p ≤ 0.05, n = 3 ± SD.

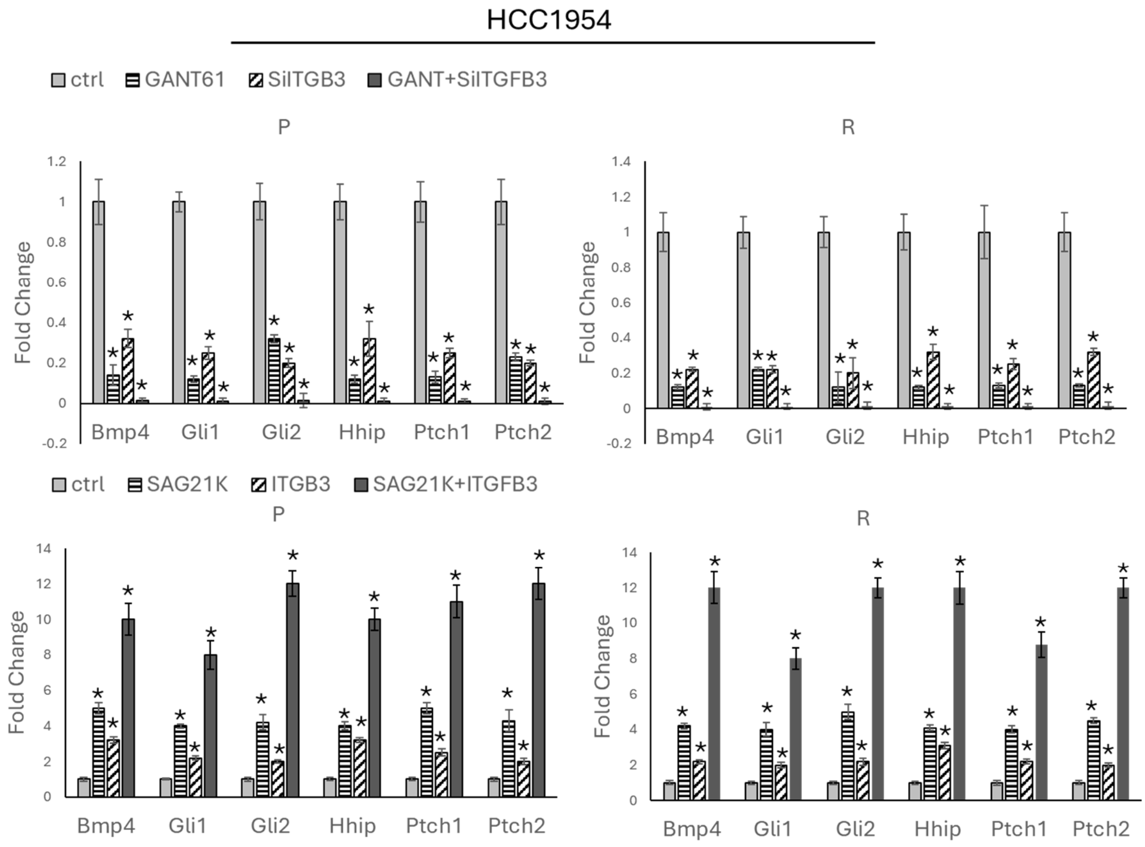

Supplementary Figure S4: Silencing of ITGB3 in the presence of GANT61 create a cumulative decrease while overexpression of ITGB3 in the presence of SAG21K create a cumulative increase in hedgehog responsive gene expressions in HCC1599 cells. A) Hedhehog responsive gene expressions analysed in the presence of GANT61 and/or ITGB3 silence in HCC1599 parental cells, B) Hedhehog responsive gene expressions analysed in the presence of GANT61 and/or ITGB3 silence in HCC1599 resistant cells C) Hedhehog responsive gene expressions analysed in the presence of SAG21K and/or ITGB3 overexpression in HCC1599 parental cells, D) Hedhehog responsive gene expressions analysed in the presence of SAG21K and/or ITGB3 overexpression in HCC1599 resistant cells. A two-way ANOVA variation test and Tukey post hoc test were used. n=3, * $p \leq 0.05$, $n = 3 \pm SD$.

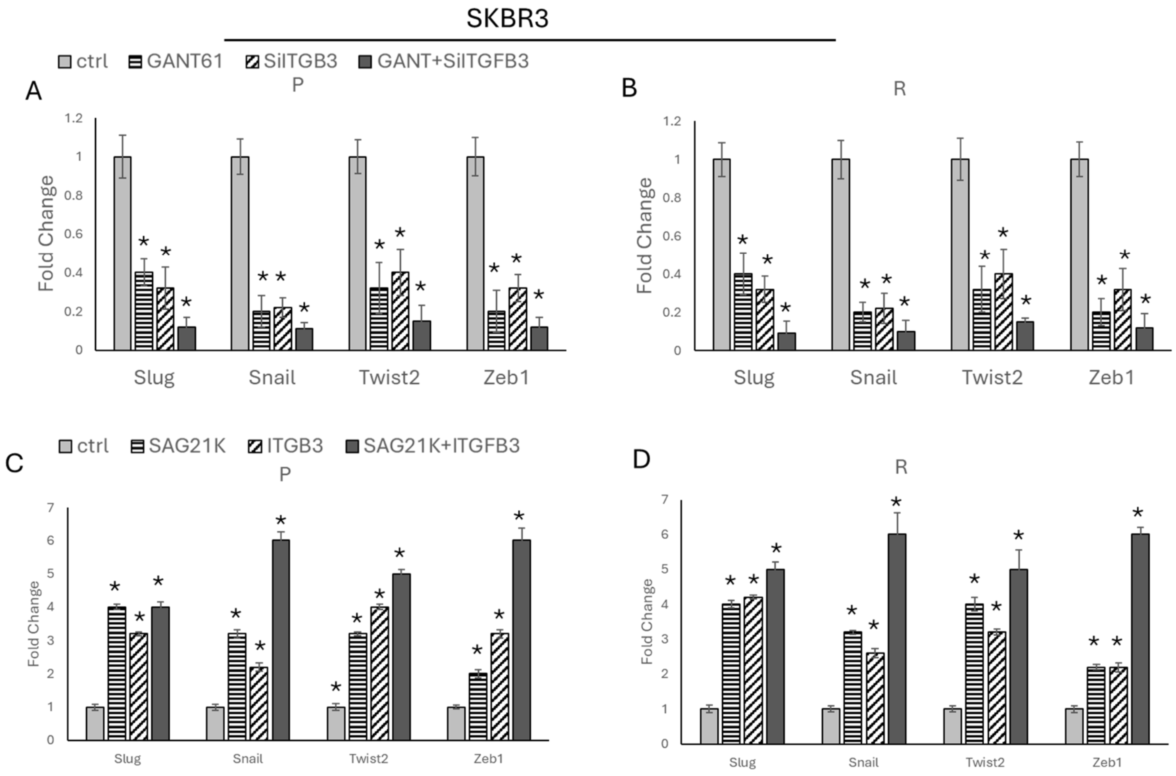

Supplementary Figure S5: Silencing of ITGB3 in the presence of GANT61 create a cumulative decrease while overexpression of ITGB3 in the presence of SAG21K create a cumulative increase in EMT responsive gene expressions in SKBR3 cells. A) EMT responsive gene expressions analysed in the presence of GANT61 and/or ITGB3 silence in SKBR3 parental cells, B) EMT responsive gene expressions analysed in the presence of GANT61 and/or ITGB3 silence in SKBR3 resistant cells C) EMT responsive gene expressions analysed in the presence of SAG21K and/or ITGB3 overexpression in SKBR3 parental cells, D) EMT responsive gene expressions analysed in the presence of SAG21K and/or ITGB3 overexpression in SKBR3 resistant cells. A two-way ANOVA variation test and Tukey post hoc test were used. n=3, * $p \leq 0.05$, n = 3 ± SD.

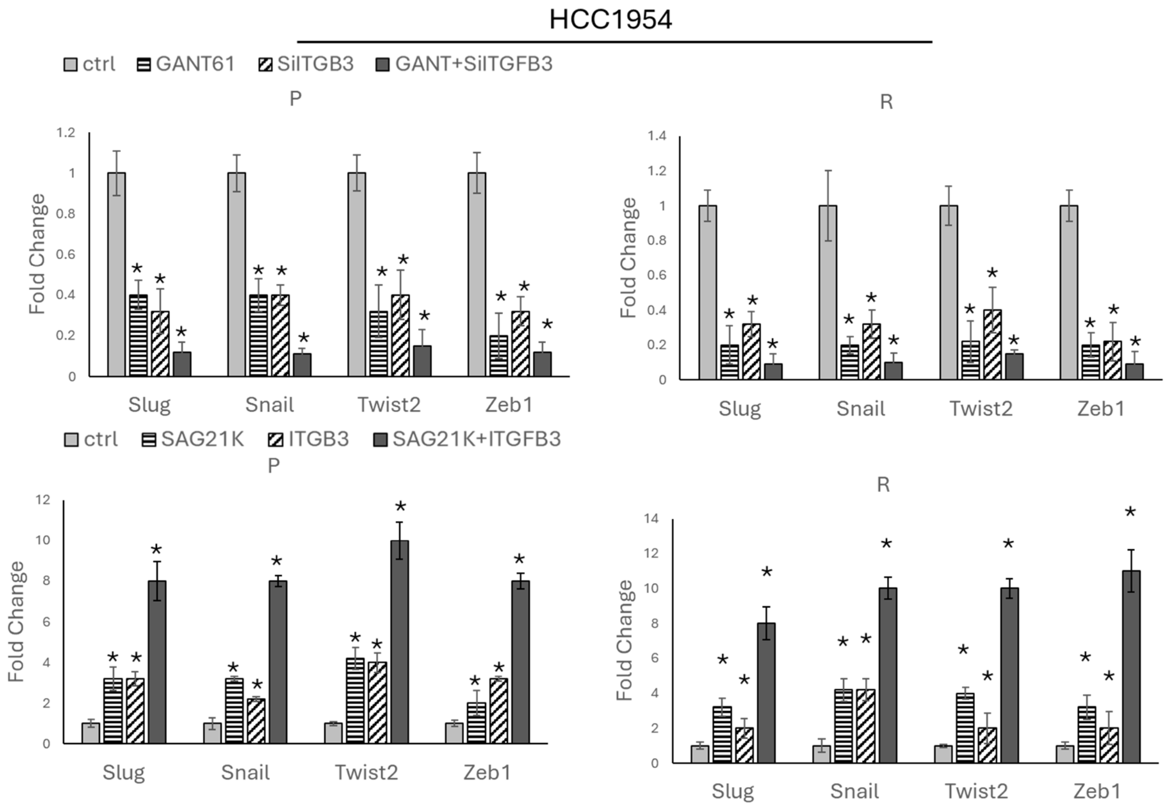

Supplementary Figure S6: Silencing of ITGB3 in the presence of GANT61 create a cumulative decrease while overexpression of ITGB3 in the presence of SAG21K create a cumulative increase in EMT responsive gene expressions in HCC1599 cells. A) EMT responsive gene expressions analysed in the presence of GANT61 and/or ITGB3 silence in HCC1599 parental cells, B) EMT responsive gene expressions analysed in the presence of GANT61 and/or ITGB3 silence in HCC1599 resistant cells C) EMT responsive gene expressions analysed in the presence of SAG21K and/or ITGB3 overexpression in HCC1599 parental cells, D) EMT responsive gene expressions analysed in the presence of SAG21K and/or ITGB3 overexpression in HCC1599 resistant cells. A two-way ANOVA variation test and Tukey post hoc test were used. n=3, * $p \leq 0.05$, n = 3 ± SD.

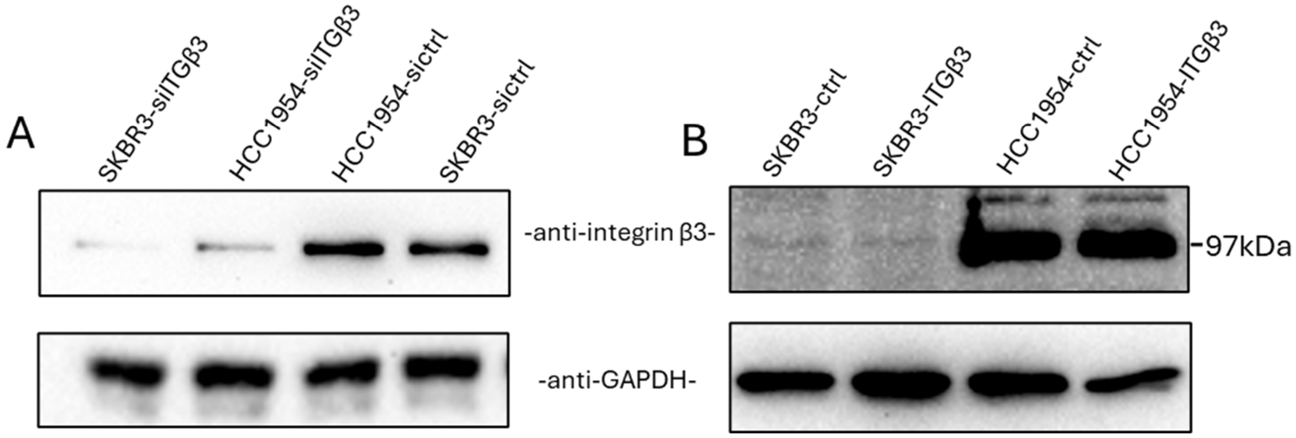

Supplementary Figure S7: Overexpression and silence of ITGβ3 in HCC1954, SKBR3 cell lines, A) western blot of silenced ITGβ3 in SKBR3 and HCC1954 cell lines, B) western blot of overexpressed ITGβ3 in SKBR3 and HCC1954 cell lines. n=3
